# *hoxa1a*-Null Zebrafish as a Model for Studying *HOXA1*-Associated Heart Malformation in Bosley–Salih–Alorainy Syndrome

**DOI:** 10.3390/biology12070899

**Published:** 2023-06-23

**Authors:** Hongjie Wang, Jingwei He, Xuemei Han, Xiuzhi Wu, Xuebin Ye, Wenchao Lv, Yao Zu

**Affiliations:** 1 International Research Center for Marine Biosciences, Ministry of Science and Technology, Shanghai Ocean University, Shanghai 201306, China; m200100110@st.shou.edu.cn (H.W.); m210100115@st.shou.edu.cn (J.H.); 1913316@st.shou.edu.cn (X.H.); wuxz1994@outlook.com (X.W.); m220100068@st.shou.edu.cn (X.Y.); m220100051@st.shou.edu.cn (W.L.); 2 Key Laboratory of Exploration and Utilization of Aquatic Genetic Resources, Ministry of Education, Shanghai Ocean University, Shanghai 201306, China; 3 Marine Biomedical Science and Technology Innovation Platform of Lin-gang Special Area, Shanghai 201306, China

**Keywords:** *hoxa1a*, heart malformation, Bosley–Salih–Alorainy syndrome, zebrafish, CRISPR

## Abstract

**Simple Summary:**

Cardiovascular disease is the number one cause of death. Congenital heart defects (CHDs) consist of 24–28% congenital malformation, yet the disease mechanism remains unclear. Zebrafish has been widely used as a model to study gene function, disease mechanism and drug discovery. It was reported that 82% of disease-causing human proteins had an obvious orthologue in zebrafish. We constructed a zebrafish disease model with *hoxa1a* mutation that exhibited heart malformation like *HOXA1*-null human patients. By studying heart disease mechanisms in zebrafish, we could further understand related diseases in humans.

**Abstract:**

Mutations in *HOXA1* can lead to diseases such as Bosley–Salih–Alorainy syndrome, involving severe cardiovascular malformations. However, the role of *HOXA1* in cardiac morphogenesis remains unclear. *hoxa1a* is a homologous gene to human *HOXA1* in zebrafish. We used CRISPR to make *hoxa1a*-null zebrafish that exhibited multiple heart malformations. In situ hybridization and sections revealed the morphological changes in mutants: enlarged ventricle with thickened myocardium and increased trabeculae, intensified OFT and inadequate heart looping, with electrocardiography supporting these pathological changes. High-speed photography captured cardiac pumping and revealed a significant decrease in cardiac output. Furthermore, lacking *hoxa1a* led to posterior body abnormality that affected movement ability, corresponding with the motor development delay in patients. Upregulation of *hox* paralogues in *hoxa1a*-null fish implied a compensatory mechanism between *hox* genes. Accordingly, we successfully constructed a *hoxa1a*-null model with a cardiac disease pattern which occurred in human *HOXA1*-associated heart malformation. The study of *hoxa1a* in zebrafish can further promote the understanding of *hox* genes and related diseases.

## 1. Introduction

Cardiac development is a precise and complex process that is strictly regulated by genetic factors and has a complex molecular regulatory network [1,2,3], but the mechanism of cardiac morphogenesis is not fully understood. The homeobox (*HOX*) family is a group of key transcription factors that regulate body axis development in vertebrates. *HOX* genes follow the characteristic of temporal–spatial collinearity expression, with 3′ end genes such as *HOXA1* being expressed first [4]. *Hoxa* genes are necessary for neural crest cell (NCC) differentiation and migration, and therefore participate in early embryonic development [5,6], whereas the role of *HOX* genes in cardiac morphogenesis is yet to be clarified.

*HOXA1*, the first expressed gene in *HOX* family, is associated with cardiac development in humans. Homozygous mutations of *HOXA1* can cause Bosley–Salih–Alorainy syndrome (BSAS) or Athabascan brainstem dysgenesis syndrome (ABDS) [7]. The characteristic symptoms include impaired eye movement, sensorineural deafness, facial and bulbar paralysis, central hypoventilation, and cardiovascular malformations [8,9]. Clinical genotyping for patients carrying cardiac malformations in BSAS indicates mutations in *HOXA1* allele [7]. Nevertheless, the mechanism of *HOXA1* influencing cardiac development remains unclear.

Several studies revealed *Hoxa1*-related symptoms in mice. *Hoxa1* in mice regulates the formation of mouse pharyngeal blood vessels and arteries. *Hoxa1* is necessary for cardiac neural crest cell migration [5]. *Hoxa1*-deficient mice exhibit defects such as interrupted aortic arch, aberrant subclavian artery, and Fallot’s tetralogy, indicating that *Hoxa1* is necessary for the formation of major arteries and cardiac outflow [10]. *Hoxa1* has been shown to have certain functional roles in neural crest cell migration as well as heart and ear development in mice [11,12]. The absence of *Hoxa1* affects cranial nerve crest cells, leading to mouse pharyngeal defects and abnormal craniofacial development [5]. The simultaneous loss of *Hoxa1* and *Hoxa*2 in mice results in skull defects and cleft palate [13]. A recent study reported bicuspid aortic valve (BAV) symptom in humans was associated with variations in the polyhistidine repeat motif of *HOXA1* [14]. *HOX* plays an important functional role in heart development in mice and humans, but the function of hoxa1 in the heart morphology and physiology associated with BSA syndrome was not fully revealed.

In summary, morphology and physiology are yet to be underlined for the understanding of cardiac pathogenesis in *HOXA1*-associated syndrome. Zebrafish have been proven to be highly suitable for studying human diseases and genetics in vertebrates [15,16]. One of the major advantages of using embryonic zebrafish is its unique optical transparency at the larval stage that allows real-time and longitudinal observation of the developing heart. Zebrafish cardiovascular physiology is also highly conserved at anatomical, cellular and membrane-biology levels [15]. For example, electrocardiography in humans and zebrafish is remarkably identical [17]. Therefore, we chose zebrafish as a model to study cardiac pathology.

CRISPR/Cas9 has been widely used for efficient and accurate disruption of gene function. Various human disease models were established in zebrafish using CRISPR/Cas9, involving the cardiovascular system and other systems. In this study, we constructed a zebrafish line with *hoxa1a (HOXA1 homology)* homozygous mutation using CRISPR/Cas9 and further observed the cardiac phenotype of the mutant. Through multidimensional observation of the cardiac phenotype, we hope to evaluate its ability as a model for studying human diseases and gain a deeper understanding of the role of *hoxa1a* in vertebrate cardiac development.

## 2. Materials and Methods

### 2.1. Zebrafish Husbandry

The wild-type zebrafish used in this study is the AB strain. The fish line was constructed and operated following the regulations on animal ethics of Shanghai Ocean University (IACUC SHOU-DW-2021-042). All fish were maintained in a UV-sterilized circulating water system at a temperature of 28.5 °C. The pH value and conductivity of the water are within normal limits, and the lighting is strictly controlled with 10 h of darkness and 14 h of light per day.

### 2.2. gRNA Synthesis

The *hoxa1a* sequence was downloaded from Ensemble (ENSDARG0000010430). gRNA was designed through Chopchop Web Tool V3 [18] and blasted through NCBI. 

To obtain double-stranded DNA fragments for in vitro transcription of gRNA, a polymerase chain reaction (PCR) system was used with a forward primer (5′-TAATACGACTCACTATANNNNNNNNNNNNNNNNNNNNGTTTTAGAGCTAGAAATAGC-3′) containing a T7 promoter upstream of the target, a standard reverse primer (5′-AAAAAAAGCACCGACTCGGTGCCAC-3′), and pUC19-scaffold plasmid as a DNA template, provided by Xiong Jingwei. The concentration of PCR products was determined by spectrophotometry (Thermofisher, Waltham, MA, USA, Nanodrop 2000c) after purification with ZYMO (D4014). gRNAs were in vitro transcribed using MAXIscript T7 kit (Ambion, Austin, TX, USA, AM1314M) and then purified by LiCl precipitation [19].

### 2.3. Microinjection and T7 Endonuclease I Assay

One-cell stage zebrafish embryo was injected with 400 pg Cas9 mRNA and 150 pg gRNA [20]. In 24 hpf, five embryos were randomly collected in one tube. A total of 15 injected embryos and 5 siblings were lysed in 50 μL 50 mM NaOH, and then neutralized with 5 μL Tris-HCl (pH = 8.0).

Prepared genome was PCR-amplified using forward primer 5′-TATCACTAGCGCCCGAACAC-3′ and reverse primer 5′-TCACAGACGATTCCACGTCC-3′.

PCR products were denatured and hybridized using a program of 95 °C 5 min; 85 °C 3 s; 25 °C 5 s. Samples were then digested by T7 endonuclease I in 37 °C for 30 min. Digested samples were subjected to agarose gel electrophoresis, and gRNA efficiency was calculated based on SURVEYOR assay [21]. To detect mutation types induced by CRISPR/Cas9, PCR products were also randomly picked for TA cloning. Single clones were picked and sequenced after colony PCR.

### 2.4. Protein 3D Structure Prediction

The nucleotide sequences from the control and mutants were translated to amino acids using Snapgene. The protein structures were predicted using the Swiss Model Tool (https://www.swissmodel.expasy.org/ accessed on 13 January 2022).

### 2.5. Visualization and Imaging

Zebrafish embryos were anesthetized in 150 mg/L Tricaine (MS-222) for 30 s (120–180 s for adults). Carefully transfer the embryos into 1% 2-hydroxyethyl agarose (Sigma-Aldrich, St. Louis, MO, USA, A9414). Put the embryos on a lateral view for microscopic imaging (fluorescence and bright-field imaging-ZEISS^®^ Axio Imager 2; High-speed imaging-C11440, Hamamatsu, Japan). The lateral view of zebrafish adults was also imaged after anesthesia by camera. Calculation of heart rate, stroke volume, cardiac output (CO) and fractional area change (FAC) were carried out as previously described [22,23].

### 2.6. Whole-Mount In Situ Hybridization (WISH)

Probe synthesis: Design probe primers in the CDS region spanning introns of the zebrafish genome. Add T7 promoter sequence 5′-TAATACGACTCACTATAGGGAGA-3′ upstream of the reverse primer. Primers used for antisense probe synthesis are listed in Supplementary Table S1. PCR products were amplified using cDNA from wild-type zebrafish as a template, and then purified for in vitro transcription (p1460, Promega, Madison, WI, USA) to synthesize the probes [24].

Hybridization: Fix zebrafish embryos. For embryos older than 48 hpf, add PTU (1-phenyl-2-thiourea) at 24 hpf to suppress melanin synthesis. Collect the embryos in a 1.5 mL centrifuge tube, remove excess water, add 1 mL of 4% paraformaldehyde, and shake overnight at 4 °C. Wash twice with 1× PBST for 5 min each time and then dehydrate using ethanol at a volume ratio of 30%, 50%, 70%, and 100%. Rehydrate using ethanol at a volume ratio of 70%, 50%, 30%, and wash twice with PBST for 5 min each time. Treat with 10 μg/mL proteinase K (Roche, Mannheim, Germany) for 20 min, and then add hybridization solution without the probe. Incubate in a hybridization oven at 65 °C for 4–5 h, followed by the addition of hybridization solution containing the probe at a concentration of 1 ng/μL, and continue to incubate overnight in the hybridization oven [24].

Blocking: Add blocking solution (prepared with 5% lamb serum and 2 mg/mL BSA) to the centrifuge tube and block the embryos at room temperature for 3–4 h. After blocking, add anti-digoxigenin antibody in a 1:5000 dilution and shake at 4 °C overnight. Wash with PBST six times at room temperature for 15 min each time. Wash with staining buffer for 5 min. Add 1 mL of NBT/BCIP to each sample, wrap in aluminum foil to avoid light and incubate until the positive signal appears blue.

After staining, the images were taken under 5× and 10× Zeiss optical microscopes. Sequences of RNA probe primers used in situ hybridization experiments will be shown.

### 2.7. Paraffin Section and Hematoxylin-Eosin (H&E) Staining

Zebrafish embryos at 3 dpf were collected in a 2 mL centrifuge tube and added with Bouin’s solution for overnight fixation at 4 °C. Remove the Bouin’s solution, rinse embryos in gradient ethanol with 70%, 80%, 90%, 95%, 100% for dehydration. Rinse embryos in 1:1 xylene: ethanol solution, and in 100% xylene twice. Add paraffin liquid to the centrifugal tube and immerse the tube in the paraffin penetration machine; the zebrafish’s abdomen was placed downward, wrapped in the wax block, made into 5 μm sections, air-dried under 37 °C and stained with hematoxylin–eosin.

### 2.8. Cartilage and Skeleton Staining

Cartilage: Samples were collected and fixed overnight at 4 °C using 4% paraformaldehyde. Afterward, wash them several times with PBST. Alcian blue (0.02%) staining solution was prepared by dissolving Alcian blue powder in a solvent comprising 80% ethanol and 20% acetic acid. Immerse samples into the staining solution for 6–24 h. After staining, wash with 100% ethanol, followed by gradient rehydration every 30 min. To remove pigments, a bleaching solution (20% H_2_O_2_ in 0.25% KOH) was used and followed by washing with PBST. Tissues were then digested with 0.1% trypsin until they became transparent, and the bones became visible. Finally, a gradient transparency process was performed using glycerol and the samples were stored in 100% glycerol.

Skeleton: After cartilage staining, prepare a 0.0002% solution of sodium alizarinsulfonate in 0.25% KOH solution, and the sample was placed in the staining solution until it turned deep purple. After removing the staining solution, the sample was gradually dehydrated using 25%, 50%, and 75% glycerol (diluted with 0.25% KOH solution), and finally 100% glycerol, until it became transparent. The sample was stored in 100% glycerol and photographed.

### 2.9. Behavior Analysis

At 6 days after fertilization, zebrafish were grouped into three categories—wild type, *hoxa1a*^+/−^, and *hoxa1a*^−/−^—to investigate their behavioral patterns. A Danio Vision system (Noldus, Wageningen, Netherlands) was used to conduct the experiment, whereby individual embryos were placed in wells containing E3 medium within a 24-well plate for 10 min of adaptation before the tests began. The behavior of each zebrafish was recorded during a 10 min exercise period. Ethovision^®^ XT 11.5 software was then utilized to analyze the data, creating digital tracks and heat maps of mobility.

### 2.10. In Vivo ECG for Adult Fish

Zebrafish were anesthetized in 168 mg/L Tricaine for 2–3 min. Electrocardiography was performed and analyzed as previously described [17,25].

### 2.11. Quantitative Real-Time PCR

The embryos of WT and *hoxa1a* mutants were collected at 3 dpf, 20 embryos each. Total RNA was extracted using Trizol reagent. A reverse-transcription kit (RR047A, TAKARA) to reverse-transcribe RNA into cDNA was used. qPCR was performed using LightCycler^®^ 480 SYBR Green (Roche, Mannheim, Germany). RT-PCR primer sequences are listed in Supplementary Table S2. Fold changes were calculated using the 2-ΔΔCt method, and statistically significant differences were defined as *p* < 0.05 using Wilcoxon’s *t*-test.

### 2.12. Statistical Analysis

All data were analyzed using GraphPad Prism 6 and one-way ANOVA was conducted for analysis of three independent groups. Mann–Whitney–Wilcoxon *t*-tests were performed for comparison between two groups. *p* > 0.5 was considered no significant difference; *p* < 0.05 significant *, *p* < 0.01, highly significant **, and *** *p* < 0.001 extremely significant. Three biological replicates were used in each experiment.

## 3. Results

### 3.1. Construction and Identification of hoxa1a Mutants

**Figure 1:**
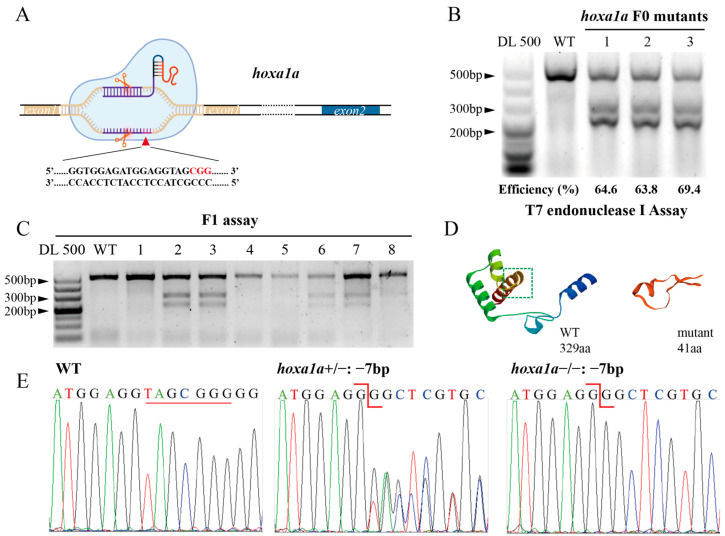
Construction of heritable *hoxa1a* mutants by CRISPR/Cas9. (**A**) Graphic illustration of CRISPR targeting the first exon in *hoxa1a* locus. (**B**) Determination of mutation efficiency by T7 Endonuclease I assay. Five founders with three replicates were examined. (**C**) Positive mutants in F1 offspring. (**D**) 3D structure prediction of normal 329aa *hoxa1a* protein and 41aa truncated mutant. (**E**) Sanger sequencing results of *hoxa1a* homozygous and heterozygous mutants.

To investigate the effect of the *hoxa1a* gene on heart development in zebrafish, we constructed a *hoxa1a* mutant using CRISPR/Cas9. As shown in Figure 1A, a single guide RNA (sgRNA) targeting exon1 of *hoxa1a* was injected into the animal pole of one-cell stage of zebrafish embryo together with Cas9 mRNA (Figure 1A). For microinjection control, we injected Cas9 mRNA with nonsense 20C gRNA and found no side effect on survival rate or heart morphogenesis (Supplementary Figure S1). Three groups of 48 hpf embryos were collected for T7E1 assay to detect gRNA efficiency (Figure 1B). The gel results of T7E1 showed that the digestion efficiency of the three groups was 64.6%, 63.8%, and 69.4%, respectively, suggesting 65.9% average efficiency. TA cloning showed that 15 (14 indel types) out of 18 alleles carried mutations after injection, and 9 out of 18 loci had frame-shift mutations (Supplementary Figure S2). Embryos (F0) were reared until sexual maturity and outcrossed with WT to obtain F1. The *hoxa1a*^+/−^ F1 was screened by T7E1 assay (Figure 1C). Two types of frame-shift mutations (−7bp and +8bp) were observed in offspring after TA cloning and sequencing (Supplementary Figure S3A). The *hoxa1a*^+/−^ F1 was incrossed, there were 1/4 WT zebrafish, 1/2 *hoxa1a*^+/−^ and 1/4 *hoxa1a*^−/−^ in the progeny, and both mutations exhibited identical cardiac malformation in F2 homozygotes. The *hoxa1a* mutant produced a premature stop codon (Supplementary Figure S3B), which truncated the protein of 41 amino acids (aa) compared with 329aa full-length homeobox a1a protein (Figure 1D). The shorter homeobox a1a protein has lost this function. Genotype identification was conducted for the progenies, and *hoxa1a*^−/−^ was identified as a frameshift mutation with a deletion of 7 bp by Sanger sequencing and peak map comparison (Figure 1E). Lacking *hoxa1a* was not lethal in zebrafish (Supplementary Figure S4). Therefore, *hoxa1a* loss-of-function zebrafish was successfully constructed.

### 3.2. Abnormal Heart Pumping and Reduced FAC in hoxa1a Homozygous Mutants

**Figure 2:**
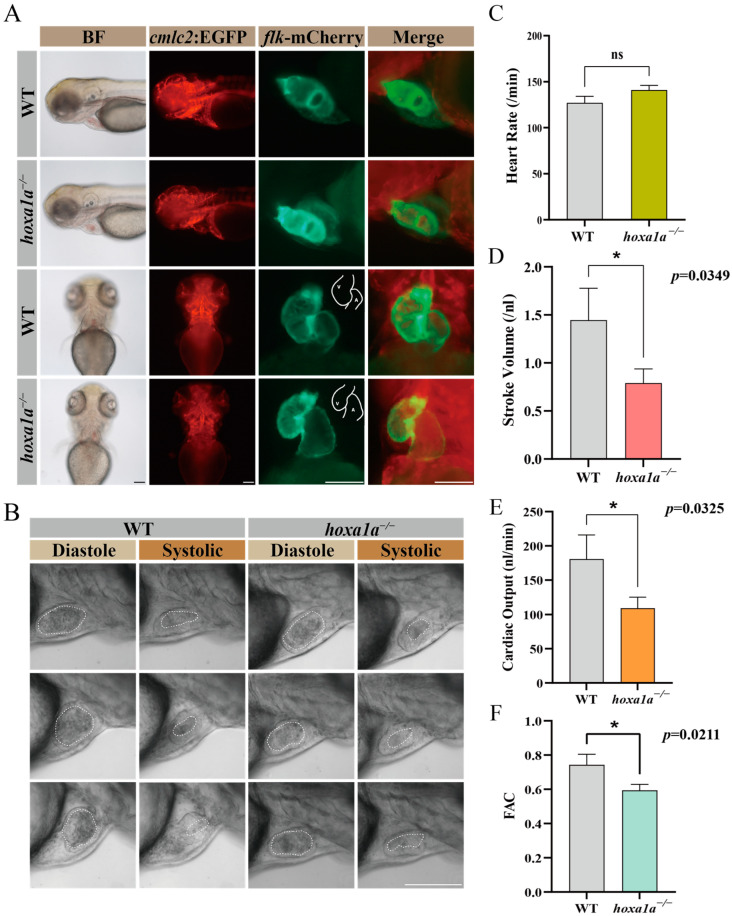
Embryo heart imaging by fluorescence and high-speed camera. (**A**) Top and lateral view of zebrafish under *cmlc2*-GFP, *flk*-mCherry and merge channel. Scale bar: 0.1 mm. (**B**) High-speed camera capturing blood flow (circled by white dashed line) and heart pumping in diastole and systole. Scale bar: 0.1 mm. (**C**–**F**) Quantified analysis data after high-speed photography using ImageJ software. Heart rate (per minute), stroke volume (nL), cardiac output (CO, nL/min) and fractional area change (FAC). Student’s *t*-test, *: *p* < 0.05, ns: no statistical difference.

To observe heart morphology in homozygous hoxa1a mutants, double fluorescence-labeled *hoxa1a* heterozygotes (myocardial cell—GFP; blood vessel endothelium—mCherry) were incrossed. Images from a normal motion camera showed a slight cardiac looping change between wild-type (WT) and *hoxa1a* homozygotes (Figure 2A). Nevertheless, after capturing the ventricular area within the endocardium under diastole and systolic using a high-speed camera (Figure 2B, Supplementary Video S1), the stroke volume was significantly reduced in mutants (Figure 2D). Heart rates remained constant (Figure 2C), whereas cardiac output and fractional area change (FAC) were severely reduced in mutants (Figure 2E,F), suggesting a weakened blood supply ability in *hoxa1a* homozygous mutants.

### 3.3. Multiple Heart Malformation Detected in hoxa1a-null Zebrafish

**Figure 3:**
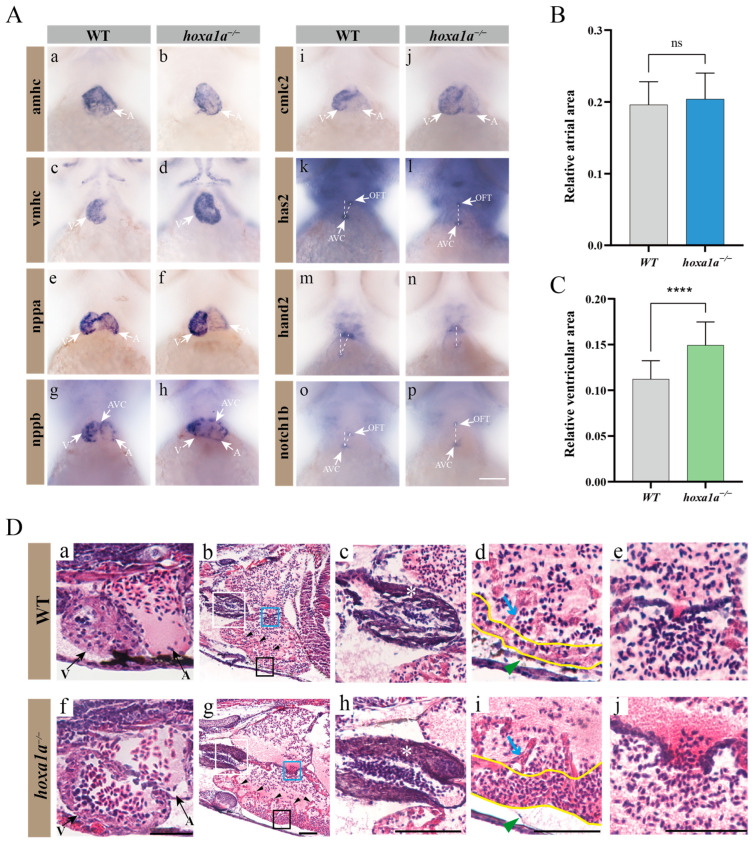
Multiple heart malformation in *hoxa1a* homozygous embryos. (**A**) In situ hybridization in 3 dpf embryos using heart labeling RNA probes—*amhc* (**a**,**b**), *vmhc* (**c**,**d**), *nppa* (**e**,**f**), *nppb* (**g**,**h**), *cmlc2* (**i**,**j**), *has2* (**k**,**l**), *hand2* (**m**,**n**), *notch1b* (**o**,**p**). (**B**) Quantified data of atrial area in (**A-b**) *amhc* and Supplementary Figure S7 (*n* = 3), Student’s *t*-test, ns: no statistical difference. (**C**) Quantified data of ventricular area in (**A-d**) *vmhc* and Supplementary Figure S8 (*n* = 3), Student’s *t*-test, ****: *p* < 0.0001. (**D**) Longitudinal sectional images stained with H&E through pericardial cavity of in *hoxa1a*^−/−^ adult mutants; see also Supplementary Figure S9. Scale bar: 0.2 mm. (**a**,**f**)-ventricle and atrium in 3 dpf embryos; (**b**,**g**)-heart morphology in 1-month-old zebrafish, areas of OFT (white squares), myocardium (black squares) and AVC (blue squares) are zoomed in, black arrows-trabeculae; (**c**,**h**)-OFT, zoomed-in images from white squares in b and g, white asterisk-OFT walls; (**d**,**i**)-myocardium, zoomed-in images from black squares in b and g, blue arrow-trabeculae, yellow wave-myocardium; (**e**,**j**)-AVC, zoomed-in images from blue squares in b and g.

To determine the cause for reduced FAC in *hoxa1a*-null zebrafish, ISH and sections were taken to further observe the exact heat chamber region and heart gene expression. The ventricle in 3 dpf *hoxa1a*^−/−^ mutants exhibited an overall enlarged shape compared to the ventricle of wild type (Figure 3(A-d), marked with a white arrow; Figure 3C; Figure 3(D-f)), while atrium remains unchanged (Figure 3(A-b); Figure 3B). Circularity of ventricle remained unchanged in mutants (Supplementary Figure S5). Enlarged ventricle in 3 dpf embryos (Figure 3(D-f)) and thickened myocardium (Figure 3(D-i), yellow lines) in one-month mutants were observed. Increased number of trabeculae was found near the muscle layer (Figure 3(D-g), labeled with black arrow). Meanwhile, outflow tract (OFT) wall was also intensified (Figure 3(D-h)). Cardiac looping angle was revealed by indirect mutual location between the atrioventricular septum (AVC) and OFT using has2 and notch1b probe. A vertical angle between AVC and OFT indicated an inadequate looping event during heart morphology (Figure 3(A-i,A-p)). *nppb* (brain natriuretic peptide) were partially mis-expressed in AVC and surrounding area in mutants, but not in wild type (Figure 3(A-g,A-h) and Supplementary Figure S6). *hoxa1a* homozygote also presented pericardial edema (Figure 3(D-h), green arrow). Therefore, lack of *hoxa1a* in zebrafish resulted in enlarged ventricle with thickened myocardium and increased number of trabeculae, intensified OFT and inadequate heart looping. Malformations such as enlarged ventricle and thickened myocardium are hallmarks of Tetralogy of Fallot, which happens in BSAS patients.

### 3.4. Prolonged QRS Wavelength and Reduced PR Interval and QTc in hoxa1a Homozygotes

**Figure 4:**
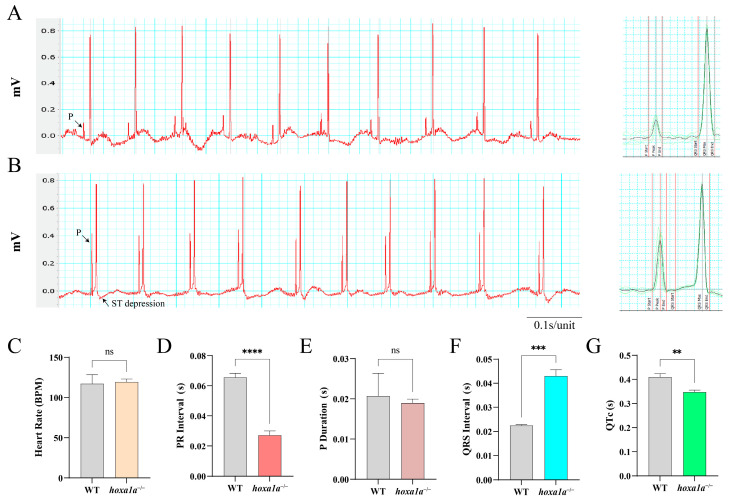
ECG analysis of adult zebrafish. Electrical signal obtained from chest muscle layer in WT (**A**) and *hoxa1a*^−/−^ (**B**), Heart rate (BPM) (**C**), PR interval(s) (**D**), P duration (s) (**E**), QRS interval(s) (**F**), QTc(s) (**G**). Student’s *t*-test, ** *p* < 0.05, *** *p* < 0.001, **** *p* < 0.0001, ns: no statistical difference.

Electrocardiograph (ECG or EKG) is a reliable method to detect cardiac abnormality. Also, the ECG wave of zebrafish and humans are remarkably similar [17,26]. We hence performed ECG in adult zebrafish (Figure 4A,B). A 0.2 s flat increasing voltage was induced first in WT and *hoxa1a* homozygotes till a sharp R wave was formed. The downshift of the ST wave in *hoxa1a* homozygotes indicated cardiac muscle damage beneath the endocardium (Figure 4B), which explained the overexpression of cardiac muscle cells in the *hoxa1a*-null ventricle. Heart rate in mutants remained constant with control (Figure 4C). PR interval was reduced, indicating atrioventricular conduction was accelerated (Figure 4D). P duration was unchanged (Figure 4E). QRS interval of the *hoxa1a*-null fish was longer than the control group, corresponding with the enlarged ventricle phenotype in Figure 3, which might cause a longer QRS interval (Figure 4F). QTc interval was significantly lower in mutants (Figure 4G). Although the above imaging did not reveal any abnormality in the *hoxa1a*-null atrium, the rising amplitude from the P wave suggested a potential lesion in the atrium region. In summary, the interpretation of the ECG diagram was consistent with the above anatomical imaging in the *hoxa1a*-null heart. Additionally, ECG excavated a faint atrium lesion that was not shown by imaging.

### 3.5. Tail Fin Malformation, Behavior Sluggishness and Craniofacial Defect in hoxa1a^−/−^ Mutants

In adult *hoxa1a* homozygotes, we observed a shrunken tail fin malformation (5/5) and one with a left twisted posterior body (Supplementary Figure S10A, signed with a red square). *hoxa1a*-null zebrafish appeared an incomplete spread and smaller spread angle in the tail bone area than the control group (Supplementary Figure S10B, marked with the red rectangle and lines).

**Figure 5:**
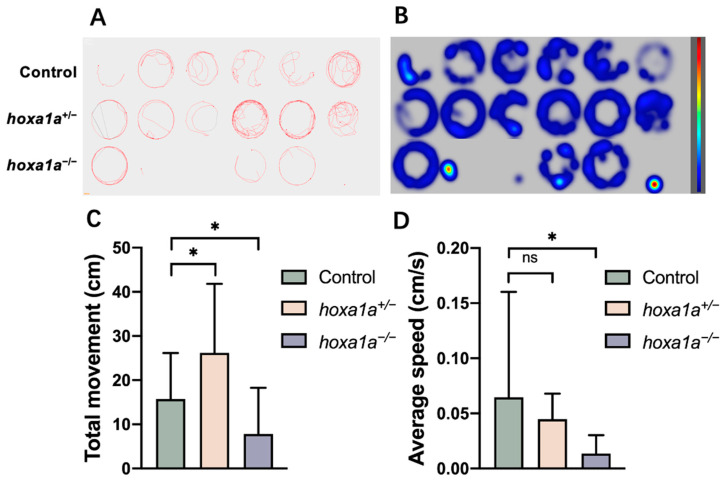
Behavior test for mobility analysis in in *hoxa1a* mutants. (**A**) Digital tracks of 6 dpf larvae from wild type, *hoxa1a*^+/−^ and *hoxa1a*^−/−^. (**B**) Heat maps of digital track A. (**C**) Total movement measurement (cm), Student’s *t*-test, *: *p* < 0.05. (**D**) Average speed measurement (cm/s), Student’s *t*-test, *: *p* < 0.05, ns: no statistical difference.

Abnormality in heart and tailbone may affect swimming ability, causing the attenuated movement; therefore, we performed a zebrafish behavior test (Figure 5A,B). Reduced total movement distance and slow swim speed were detected in *hoxa1a* homozygotes in 6 dpf (Figure 5C, D), and some embryos exhibited movement reluctance (Figure 5A, line 3, no. 2, 3 and 6), suggesting a weakened movement capability due to the malformation on the tail fin. Average speed control revealed no difference from wild type to *hoxa1a* heterozygotes (Figure 5D), while *hoxa1a* heterozygotes showed more total movement (Figure 5C). In sum, *hoxa1a* influenced tail development in zebrafish and the absence of *hoxa1a* caused tail malformation.

Another bone defect related to craniofacial development was observed in juvenile *hoxa1a* homozygotes. *hoxa1a* homozygote at 1 month showed a loss of cartilage at the anterior end of the first pharyngeal arch (black arrow, Supplementary Figure S10C). This symptom was also reported in human ABDS and BSAS patients as facial malformation [7].

### 3.6. hoxa1a Homozygotes Occurred in Gene Compensation and Upregulated Paralogous hox Genes

**Figure 6:**
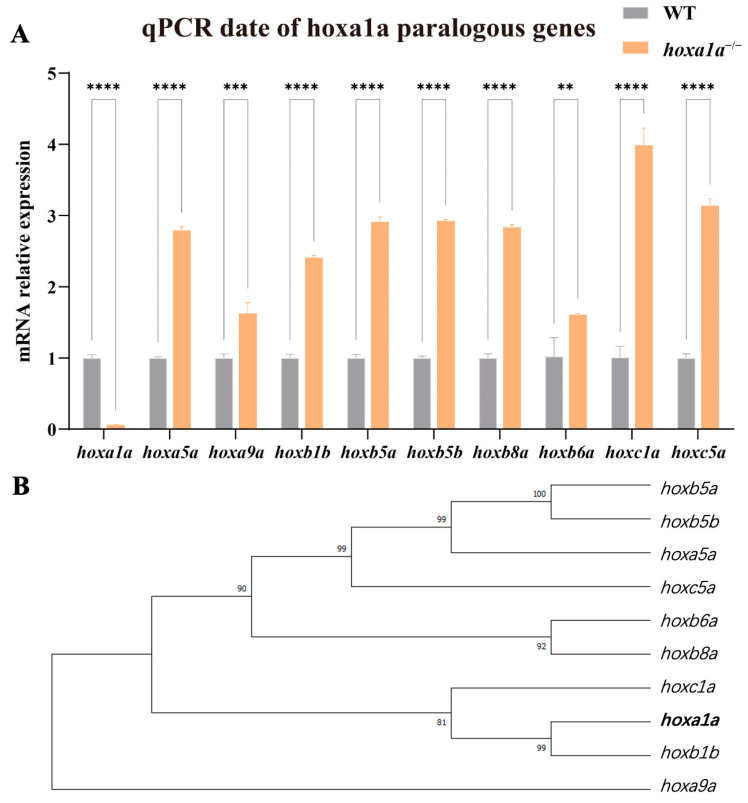
*hoxa1a* paralogous regulation. (**A**) Results of qPCR for paralogous genes in the *hox* gene cluster in the embryonic zebrafish. (Student’s *t*-test, ** *p* < 0.05, *** *p* < 0.001, **** *p* < 0.0001). (**B**) Phylogenetic tree showing relationships between *hoxa1a* and paralogous *hox* genes in zebrafish.

*hoxa1a* homozygous zebrafish appear to have less severe cardiac phenotypes than those in mice [10]. A possible explanation is that zebrafish have seven *hox* clusters (four in mice) and these genes compensate mutually, while one is missing. *Hox* family shares a conservative DNA-binding motif [27] and thereby are highly similar (Supplementary Figure S11). Therefore, we hypothesized paralogous genes may compensate for *hoxa1a* function to a certain extent. We conducted qPCR to examine the expression of *hoxa1a* paralogous genes. Now that *hoxa1a* was fully truncated and had a loss of function, paralogous genes from *hoxaa*, *hoxba*, *hoxbb* and *hoxca* clusters were all significantly upregulated 1.5- to 4-fold (Figure 6A). Although *hoxa1a* and *hoxb1b* clustered into one branch in the phylogenetic tree (Figure 6B), *hoxc1a* represented the biggest upregulation among the tested paralogous genes. Overall, these data indicate the existence of a compensation effect between *hox* genes.

## 4. Discussion

In this study, a homozygous mutant zebrafish lacking *hoxa1a* appeared with multiple heart malformations, similar to patients with *HOXA1* mutations accompanied by congenital heart defects such as dilated heart chamber, Tetralogy of Fallot and septum defects [10]. Using various visualization methods (fluorescence, high-speed imaging, section, and in situ hybridization), we discovered malformations including enlarged ventricle with thickened myocardium and increased number of trabeculae, intensified OFT and inadequate heart looping. The cardiac output ability in zebrafish mutants was assessed using a high-speed camera, and the FAC indicated that the abnormal ventricle led to reduced pumping ability (as evidenced by electrophysiology). All the abnormal development in zebrafish *hoxa1a* mutants suggests *hoxa1a* is essential for heart development. Therefore, *hoxa1a*-null zebrafish is an identical model for studying heart malformation in patients with *HOXA1* mutation. Meanwhile, the similar heart disease pattern between zebrafish and humans further illustrated zebrafish is suitable for human disease study.

We observed that the homozygous ventricles exhibited enlargement, accompanied by an increase in myocardial thickness, and the sections also showed an increase in trabeculae in the ventricle. Observations in the in situ hybridization images revealed a slight change in the angle of cardiac looping, which may be the reason for the ventricular malformation. Due to the changes in the ventricle, fractional area change was significantly lower than that of the wild type. These pathological changes accumulated in the heart, resulting in an overwhelmingly thick myocardium in the heart of adult fish. The enlargement in the ventricle and thickened myocardium may be due to genetic mutations in *hoxa1a*, and cardiac malformation could accumulate when maturing. Heart looping abnormality in zebrafish indicated lacking *hoxa1a* affects migration of heart progenitor [6]. The increase in ventricular muscle trabeculae matched the symptom of noncompaction of the ventricular myocardium (NVM) [28].

Apart from heart malformation in *hoxa1a*-null zebrafish, we also observed a tail abnormality. This posterior disability may affect movement ability, since the tail fin (or caudal fin) provides a thrust force when hunting or escaping [29]. Meanwhile, considering *hoxa1a*-null fish carrying multiple heart malfunctions such as low cardiac output and movement capability were further restricted compared to the wild type. This movement disorder in fish also happens in BSAS patients with *HOXA1* mutations who present motor development delay [8].

The qPCR analysis in *hoxa1a* homozygotes revealed significant upregulation from paralogous *hox* genes, and *hoxc1a* showed the biggest upregulation (fourfold), implying a potential direct compensation response to *hoxa1a* missing. Due to the homology of the *hox* genes, they all have a homologous protein region encoded by a repeated sequence, which may compensate functionally for the effect of *hoxa1a* deficiency. Further research could focus on the mutual regulation among the *hox* family and determine whether this event is independent in bony fish that experience an extra chromosomal duplication than mammals [30].

In conclusion, the heart development and disease patterns with *HOX* mutation in zebrafish and humans are highly similar. Future disease studies on BSAS, ABDS or BAV could use *hoxa1a*-null zebrafish as an ideal model for investigating disease mechanisms, while screening potential drugs for alleviating heart malformation.

## 5. Conclusions

A heritable *hoxa1a*-null zebrafish line was successfully constructed. Multidimensional cardiac measurements and imaging revealed similar heart malformations between human and zebrafish with *HOXA1* and *hoxa1a* mutations, including enlarged ventricle with thickened myocardium and increased number of trabeculae, intensified OFT and inadequate heart looping. This ideal animal model will provide a new understanding of *BSAS* and help in discovering relevant treatments.

## Data Availability

All data needed to evaluate the conclusions in the paper are included in the paper and/or the Supplementary Materials. The plasmid system and fish lines used in this study are available from the corresponding author upon reasonable request.

## Supplementary Materials

The following supporting information can be downloaded at https://www.mdpi.com/article/10.3390/biology12070899/s1. Table S1: Primers for ISH probe synthesis; Table S2: Primers for qPCR; Figure S1: Cas9 mRNA dosage response; Figure S2: Eighteen sequenced *hoxa1a* alleles after injection; Figure S3: Two frame-shift (−7 bp and +8 bp) mutation types isolated from F1 progeny; Figure S4: Survival rate of wild-type siblings, *hoxa1a* heterozygotes and *hoxa1a*-null zebrafish during embryo stage; Figure S5: Quantified ventricle circularity; Figure S6: In situ hybridization using *nppb* probes labeling heart chambers in 3 dpf embryos; Figure S7: In situ hybridization using *amhc* probes labeling atrium in 3 dpf embryos; Figure S8: In situ hybridization using *vmhc* probes labeling ventricle in 3 dpf embryos; Figure S9: Longitudinal sectional images stained with H&E between WT control and *hoxa1a*^−/−^ mutants in 1-month-old zebrafish with different slices; Figure S10: *hoxa1a*-null zebrafish exhibited abnormal tail fin phenotype and craniofacial development; Figure S11: Phylogenetic tree showing relationships between zebrafish *hoxa1a* and homologous/paralogous genes in other species; Video S1: High-speed imaging in the heart of a *hoxa1a*^−/−^ mutant and a wild-type fish (3 dpf).
