# Supplementary material for: hoxa1a-Null Zebrafish as a Model for Studying HOXA1-Associated Heart Malformation in Bosley–Salih–Alorainy Syndrome
Source: Biology (Basel). 2023 Jun 23;12(7):899. doi: 10.3390/biology12070899 (PMC10376578; doi:10.3390/biology12070899)
Supplement: Supplementary file 1 [file biology-12-00899-s001.zip › Revised Supplementary material.pdf]

Supplementary materials for

hoxa1a null zebrafish as a model for studying *HOXA1*-associated heart malformation in Bosley-Salih-Alorainy Syndrome

Hongjie Wang [1,2,†], Jingwei He [1,2,†], Xuemei Han [1,2,†], Xiuzhi Wu [1,2], Xuebin Ye [1,2,3], Wenchao Lv [1,2], and Yao Zu [1,2,3,*]

1 International Research Center for Marine Biosciences, Ministry of Science and Technology, Shanghai Ocean University, Shanghai 201306, China;

2 Key Laboratory of Exploration and Utilization of Aquatic Genetic Resources, Ministry of Education, Shanghai Ocean University, Shanghai 201306, China.

3 Marine Biomedical Science and Technology Innovation Platform of Lin-gang Special Area, Shanghai 201306, China

† These authors contributed equally to this work

* Correspondence: yzu@shou.edu.cn; Tel.: +86-21-61900474

Supplemental Item	Title
Table S1	Primers for ISH probes synthesis
Table S2	Primers for qPCR
Figure S1	Cas9 mRNA dosage response.
Figure S2	18 sequenced *hoxa1a* alleles after injection.
Figure S3	Two frameshift (-7bp and +8bp) mutation types isolated from F1 progeny.
Figure S4	Survival rate of wild type siblings, *hoxa1a* heterozygotes and *hoxa1a* null zebrafish during embryos stage.
Figure S5	Quantified ventricle circularity.
Figure S6	In-situ hybridization using *nppb* probes labeling heart chambers in 3dpf embryos.
Figure S□	In-situ hybridization using *amhc* probes labeling atrium in 3dpf embryos.
Figure S8	In-situ hybridization using v*mhc* probes labeling ventricle in 3dpf embryos.
Figure S9	Longitudinal sectional images stained with H&E between WT control and *hoxa1a*$^{-/-}$ mutants in 1-month-old zebrafish with different slices.
Figure S10	*hoxa1a* null zebrafish exhibited abnormal tail fin phenotype and craniofacial development.
Figure S11	Phylogenetic tree showing relationships between zebrafish *hoxa1a* and homologous/paralogous genes in other species.

Table S1. Primers for ISH probes synthesis

Locus	Forward primer (5’-3’)	Reserve primer (5’-3’)
amhc	TAATACGACTCACTATAGGGAGATAATGCATGATAGCGCCGGT	GGAGTACGTGAAGGGGCAAA
vmhc	TAATACGACTCACTATAGGGAGAAGAGTCCCACCACAGTCTCA	CCTCTCTCAGCGGAAACCTG
nppa	TAATACGACTCACTATAGGGAGAAGGGTGCTGGAAGACCCTAT	GAGACACTCAGAGATGGCCG
nppb	CATTCCCGTAGTCGGCCTTC	GGATCCATTAACCCTCACTAAAGGGAACCCCGACTGTGTTACATCCCAA
cmlc2	TTGGCTGCATAGATCAGAACC	TAATACGACTCACTATAGGGAGGCTGCTGATGTGAATGTTGAAC
has2	TAATACGACTCACTATAGGGAGACGCGCGGTGTATTTCGTGGC	GGCCCTATGCATCGCAGCCT
hand2	ACCATGGCACCTTCGTACAG	TAATACGACTCACTATAGGGAGATGGCCAACCAGTTCTCCCTTT
notch1b	GAGGTGGCCGCCAAACAGCA	CCCACATGCGTGAGCGTCGT

Table S2. Primers for qPCR

Locus	Forward primer (5'-3')	Reserve primer (5'-3')
hoxa5a	CGTGCTCATCCTTAGCCAACTCTC	CTGTGAAGCTGTCTGTCCACTACC
hoxa9a	AATGATGACGGCACGGAATA	TCGCATGAAGCCAGTTGGA
hoxb1b	TCGGTCTCTGCAAGCCACTATCA	TCTTGGAGTCGCTGTCACTGTCA
hoxb5a	AGGCGTCTCACAGAGCGAACA	TGCCGTCTGGTCCAGTCATATCAT
hoxb5b	AAGCAAGAATCTGTGGCGACCTC	GCGTCTGGTAGCGAGTGTAAGC
hoxb8a	CCATGTGCGGTAACCTGTC	GTCGCTATACTGAACCAAGTCG
hoxb6a	CCCTTGGATGCAACGGATGA	CGAAGACGCCGCATTGAGA
hoxc1a	CCTCCTTCCAAAGCTGTCGT	CCTCGTCAGAATCTCGTCCG
hoxc5a	TCAGTCACGGACTGTTGAGC	CGACGTGTGAGGTATCGGTT

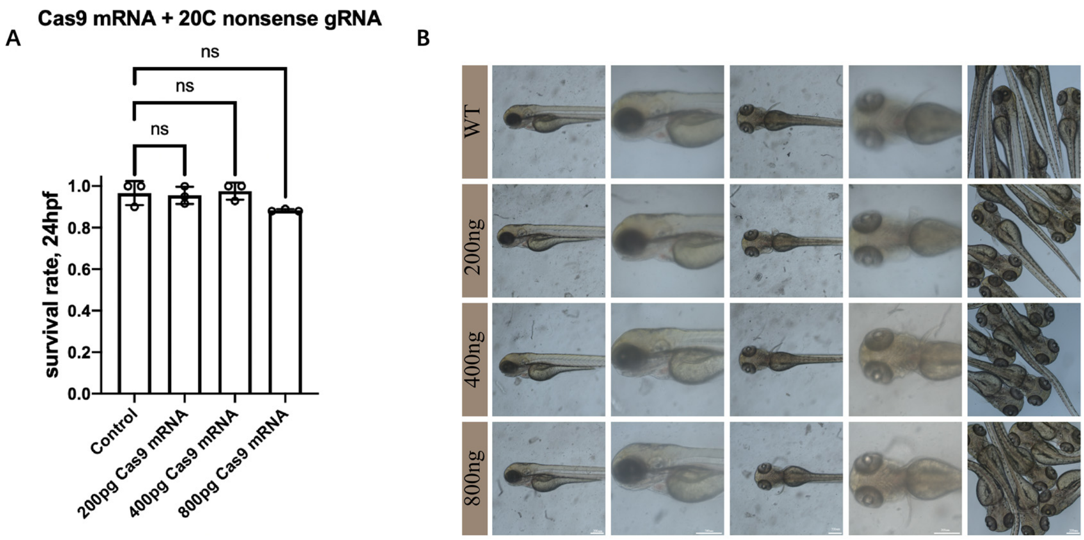

Figure S1. Cas9 mRNA dosage response. (A) Survival rate in 24hpf embryos after microinjected into 200pg/400pg/800pg Cas9 mRNA with 150pg 20C nonsense gRNA. (B) Overall view and heart imaging after co-injection of Cas9 mRNA and 20C nonsense gRNA.

18 sequenced *hoxa1a* alleles after gRNA 1 injection

ATTTTTCGTCCATAAGTGGTGGTGGAGATGGAGGTAGCGGGGCTCGTGCTCAGTCAGGGCGT (Reference)

---CTCAGTCAGGGCGT -92*

---CTCAGTCAGGGCGT -62*

ATTTTTCGTCCATAAGTGGTG--------------------GGCTCGTGCTCAGTCAGGGCGT -20*

ATTTTTCGTCCATAAGTGGTGG---AG--------------GGCTCGTGCTCAGTCAGGGCGT -17*

ATTTTTCGTCCATAAGTGGTGGTGG----------------GGCTCGTGCTCAGTCAGGGCGT -16*

ATTTTTCGTCCATAATTGGTGGTGGAGATGGAGGTAG----------TGCTCAGTCAGGGCGT -10*

ATTTTTCGTCCATAAGTGGTGGTGGAGATGGAGG---------TTCGTGCTCAGTCAGGGCGT -9

ATTTTTCGTCCATAAGTGGTGGTGGAGATGGAGG------TAGCTCGTGCTCAGTCAGGGCGT -6

ATTTTTCGTCCATAAGTGGTGGTGGAGATGGAGGTAG--GGGGCTCGTGCTCAGTCAGGGCGT -2*

ATTTTTCGTCCATAAGTGGTGGTGGAGATGGAGGTAGGGGGGGCTCGTGCTCAGTCAGGGCGT 0

ATTTTTCGTCCATAAGTGGTGGTGGAGATGGAGATGGAGGGGGCTCGTGCTCAGTCAGGGCGT 0

ATTTTTCGTCCATAAGTGGTGGTGGAGATGGAGGTAGctCGGGGGCTCGTGCTCAGTCAGGGCGT +2* （x2）

ATTTTTCGTCCATAAGTGGTGGTGGAGATGGAGGTAgctCGGGGGCTCGTGCTCAGTCAGGGCGT +3

ATTTTTCGTCCATAAGTGGTGGTGGAGATGGAGGTGGagatggagaCGGGGGCTCGTGCTCAGTCAGGGCGT +9

ATTTTTCGTCCATAAGTGGTGGTGGAGATGGAGGTAGCGGGGGCTCGTGCTCAGTCAGGGCGT 0, Wild type (x3)

Indel mutations: 15 out of 18 (83.3%)

*Frame-shift mutations: 9 out of 18 (50%)

Insertions: 4 out of 18

e.g., +2bp peak map

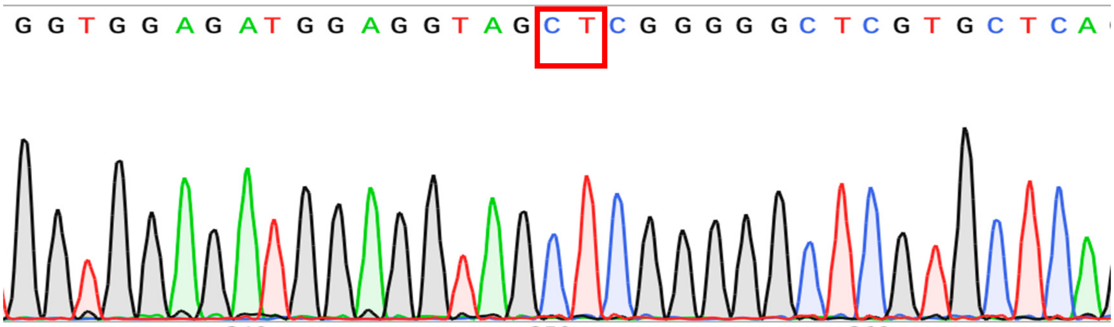

Deletions: 9 out of 18

e.g., -10bp

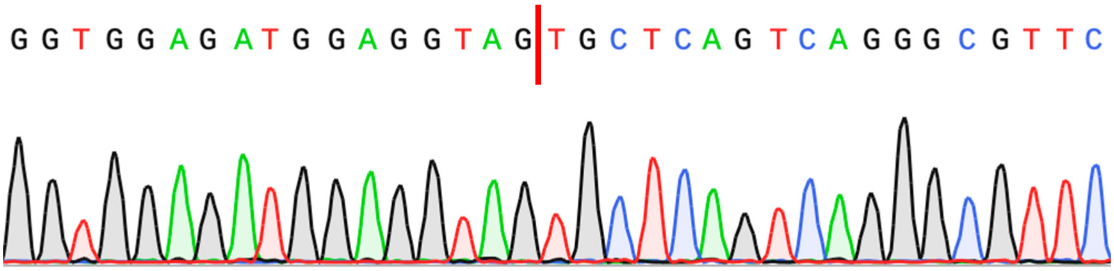

Single-site mutations: 4 out of 18

e.g., C to G conversion

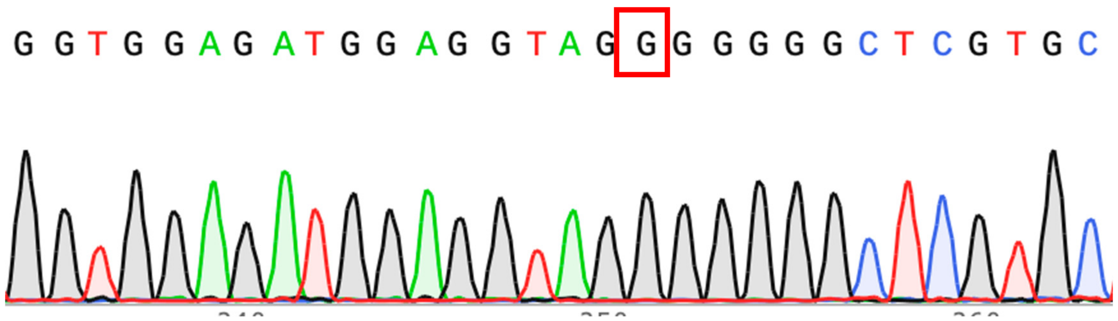

Figure S2. 18 sequenced *hoxa1a* alleles after injection. 15 out of 18 alleles carried mutations (including small insertion/deletion and single-site mutations). 9 out of 18 alleles carried frame-shift mutations (marked with *).

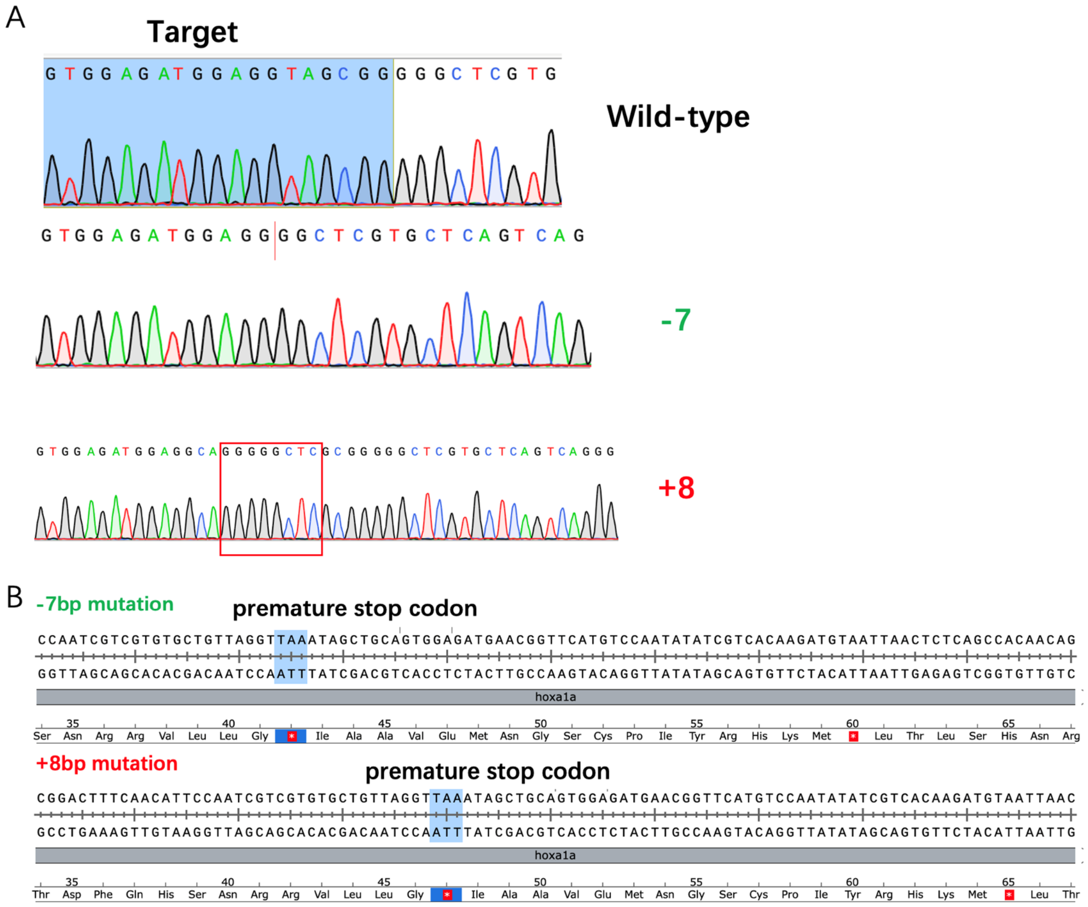

Figure S2. Two frameshift (-7bp and +8bp) mutation types isolated from F1 progeny. (A) Peak map after TA cloning. (B) Premature stop codon in -7bp mutation (42aa) and +8bp mutation (47aa).

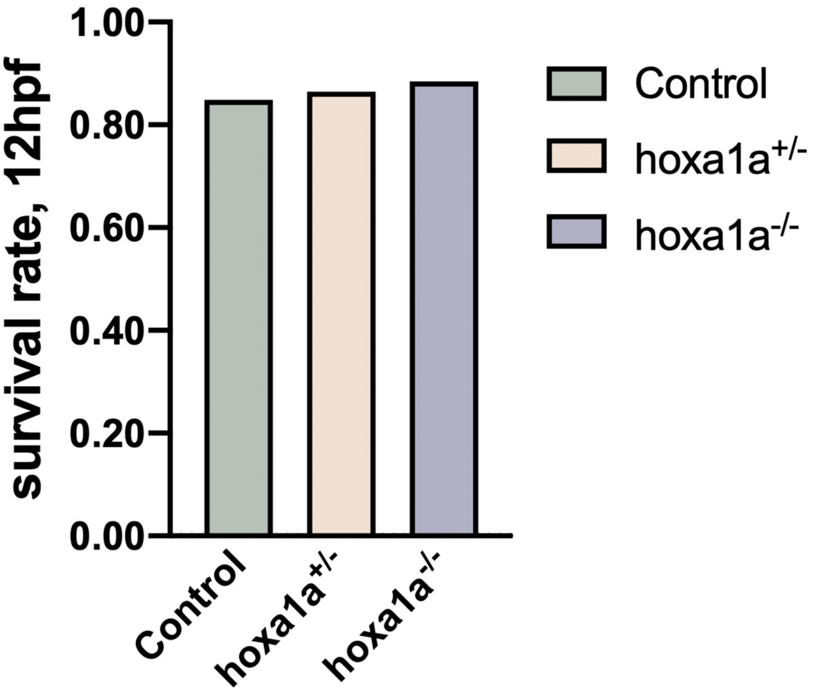

Figure S3. Survival rate of wild type siblings, *hoxa1a* heterozygotes and *hoxa1a* null zebrafish during embryos stage.

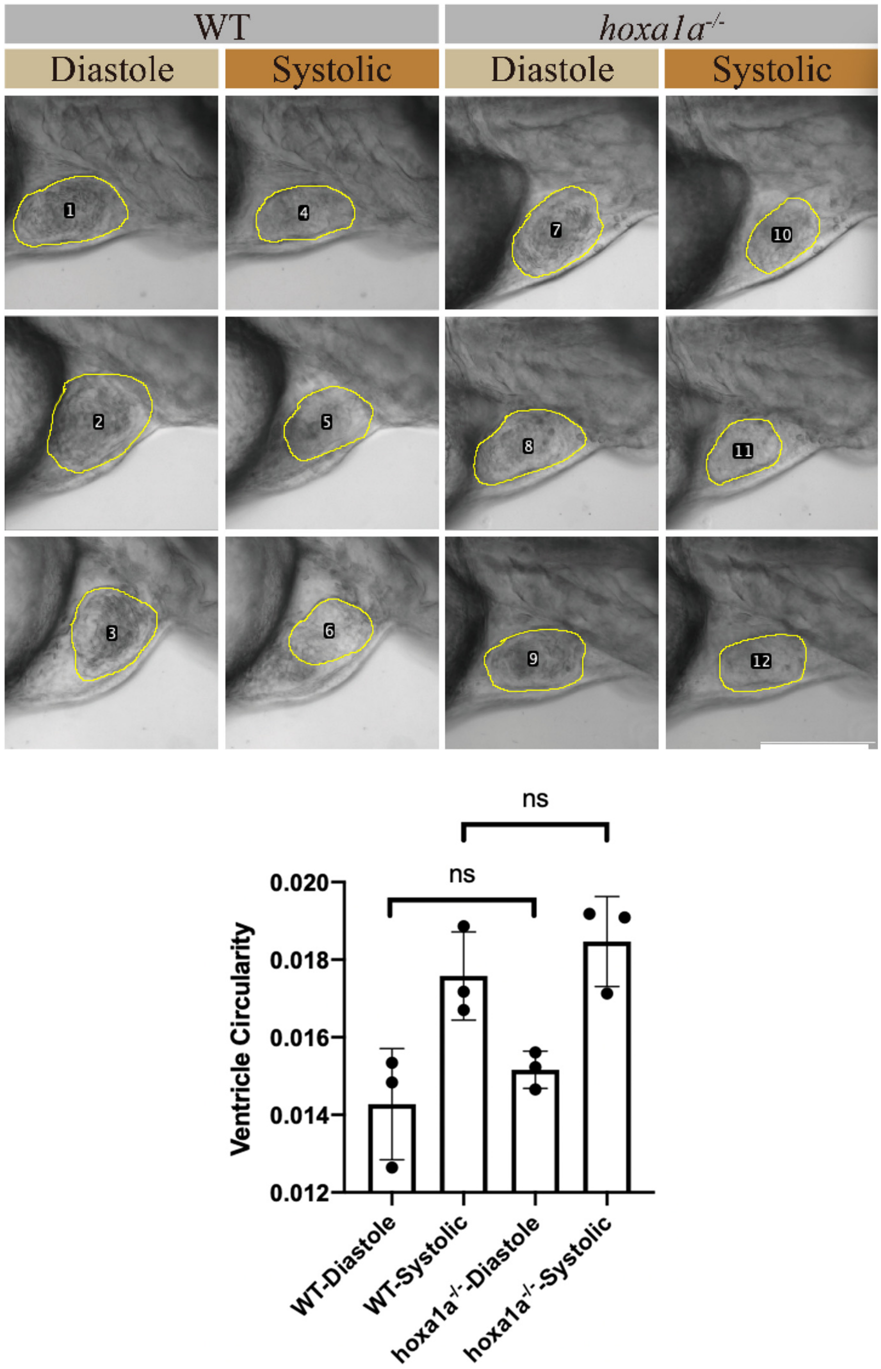

Figure S4. Quantified ventricle circularity. Circularity $= \frac{4\pi \times \text{area}}{\text{perimeter}^2}$. There is no significant difference between WT and hoxa1a homozygotes in diastole and systolic.

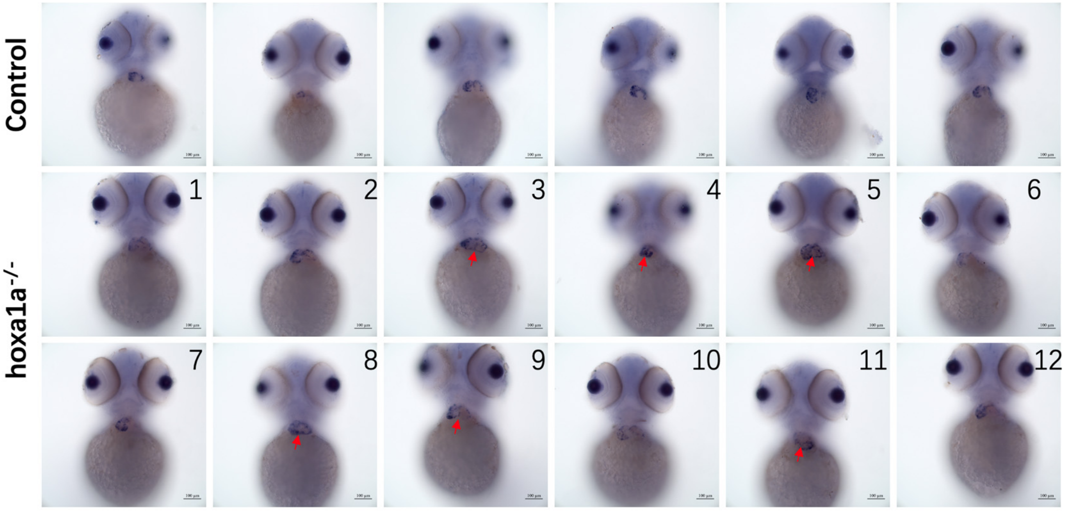

Figure S5. In-situ hybridization using *nppb* probes labeling heart chambers in 3dpf embryos. *nppb* mis-expressed in 3, 4, 5, 8, 9 and 11 in *hoxa1a* homozygotes.

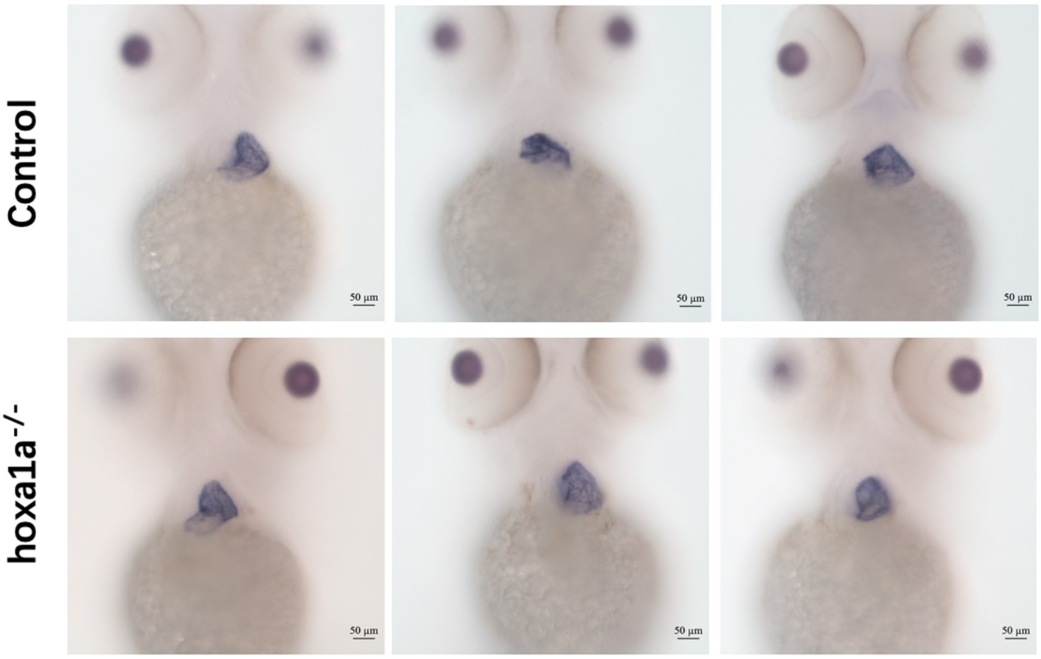

Figure S6. In-situ hybridization using *amhc* probes labeling atrium in 3dpf embryos.

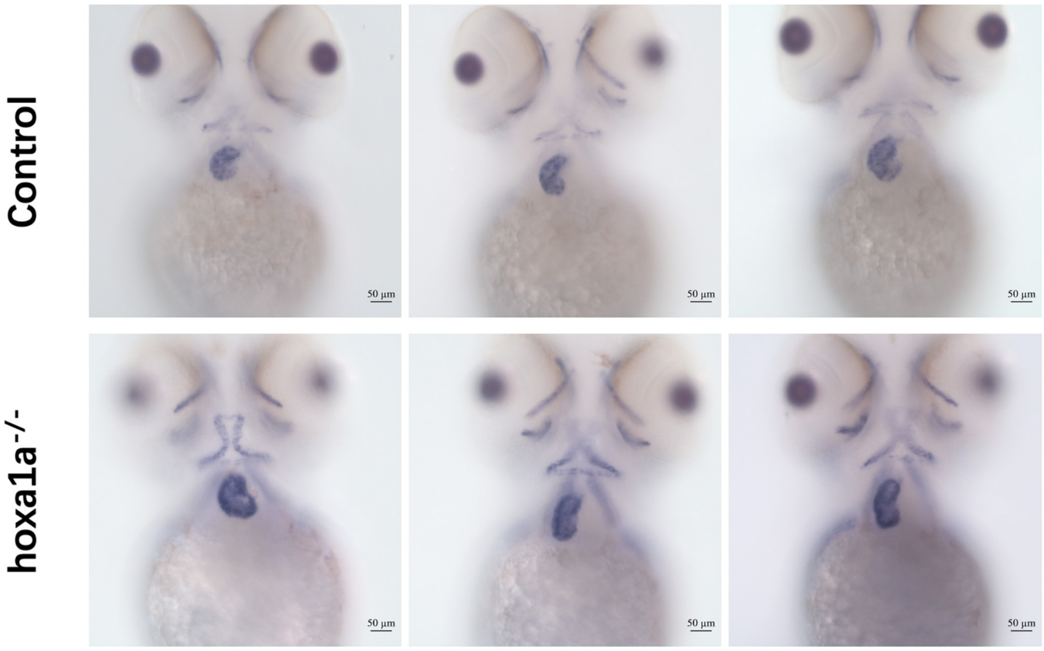

Figure S□. In-situ hybridization using v*mhc* probes labeling ventricle in 3dpf embryos.

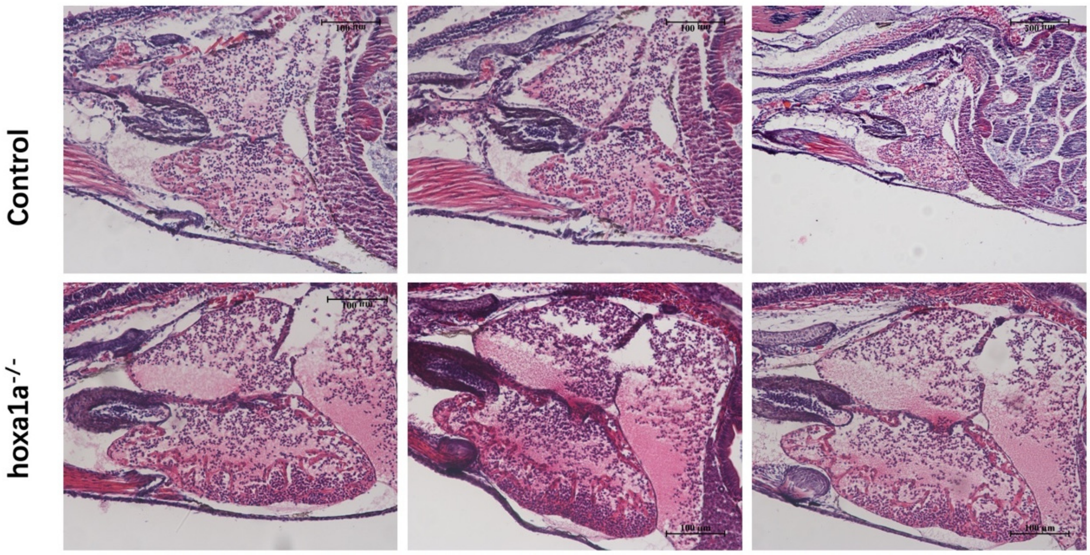

Figure S8. Longitudinal sectional images stained with H&E between WT control and *hoxa1a*$^{-/-}$ mutants in 1-month-old zebrafish with different slices.

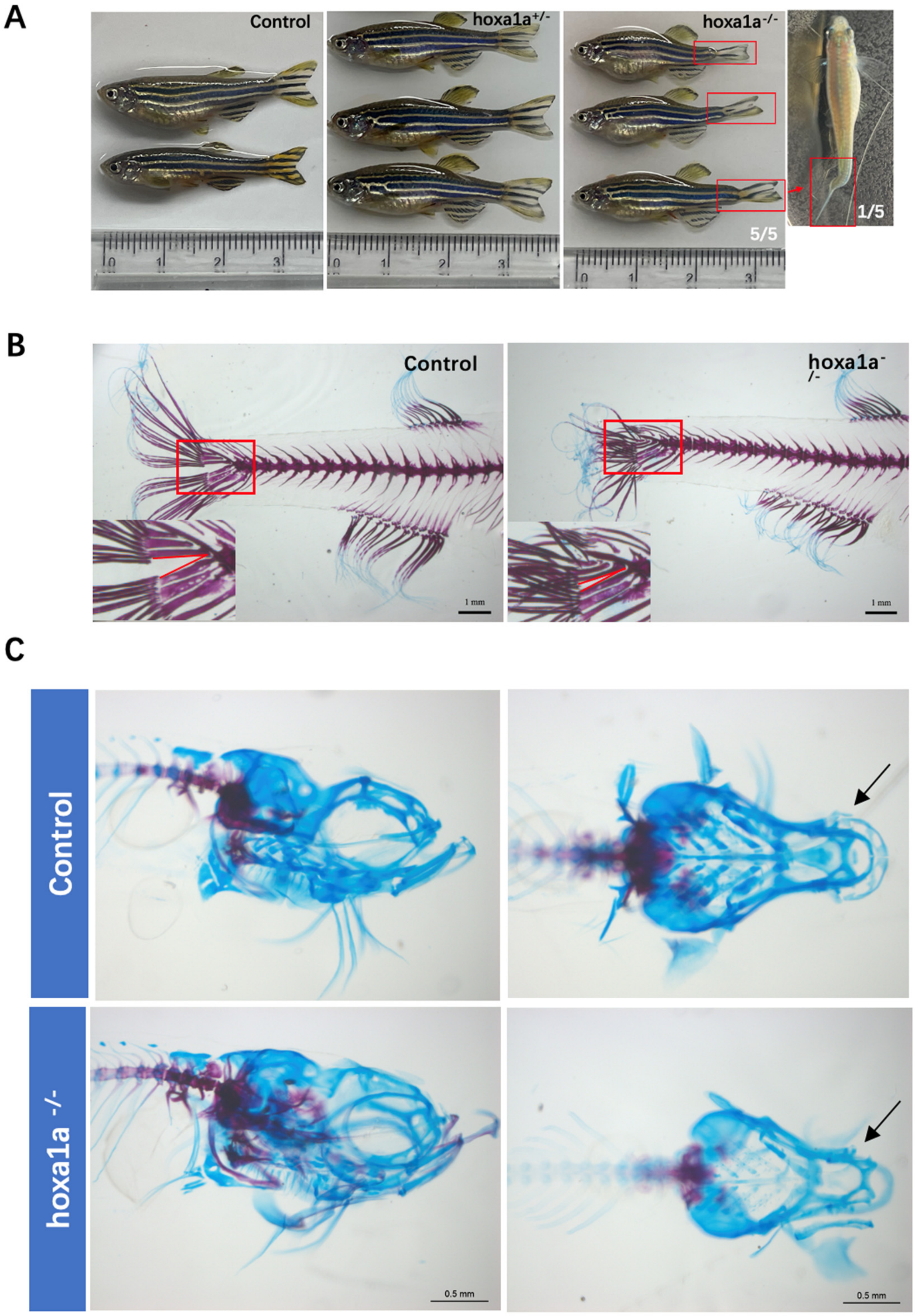

Figure S9. *hoxa1a* null zebrafish exhibited abnormal tail fin phenotype and craniofacial development. (A) *hoxa1a* homozygotes exhibited smaller and insufficiently spread tail fin compared to wild type and heterozygotes. (B) Skeleton and cartilage staining in one month fish. Tail bones of *hoxa1a* homozygotes had a smaller spread angle (marked with red lines). Scale bar: 1mm. (C) Cartilage staining of wild-type and *hoxa1a* homozygous juvenile zebrafish at 1 month. *hoxa1a* homozygote at 1 month showed a loss of cartilage

at the anterior end of the first pharyngeal arch (black arrow). Scale bar: 0.5mm.

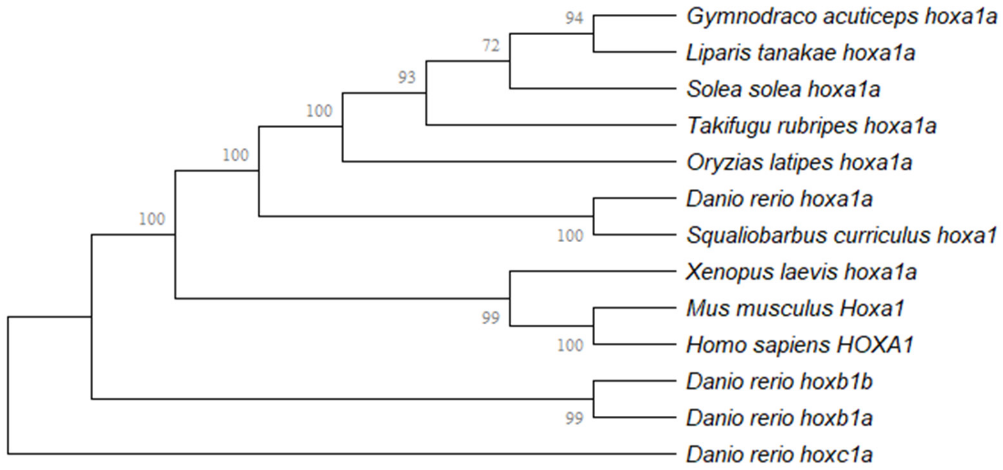

Figure S10. Phylogenetic tree showing relationships between zebrafish *hoxa1a* and homologous/paralogous genes in other species.
