# Supplementary figures and images for: hoxa1a-Null Zebrafish as a Model for Studying HOXA1-Associated Heart Malformation in Bosley–Salih–Alorainy Syndrome
Source: Biology (Basel). 2023 Jun 23;12(7):899. doi: 10.3390/biology12070899 (PMC10376578; doi:10.3390/biology12070899)

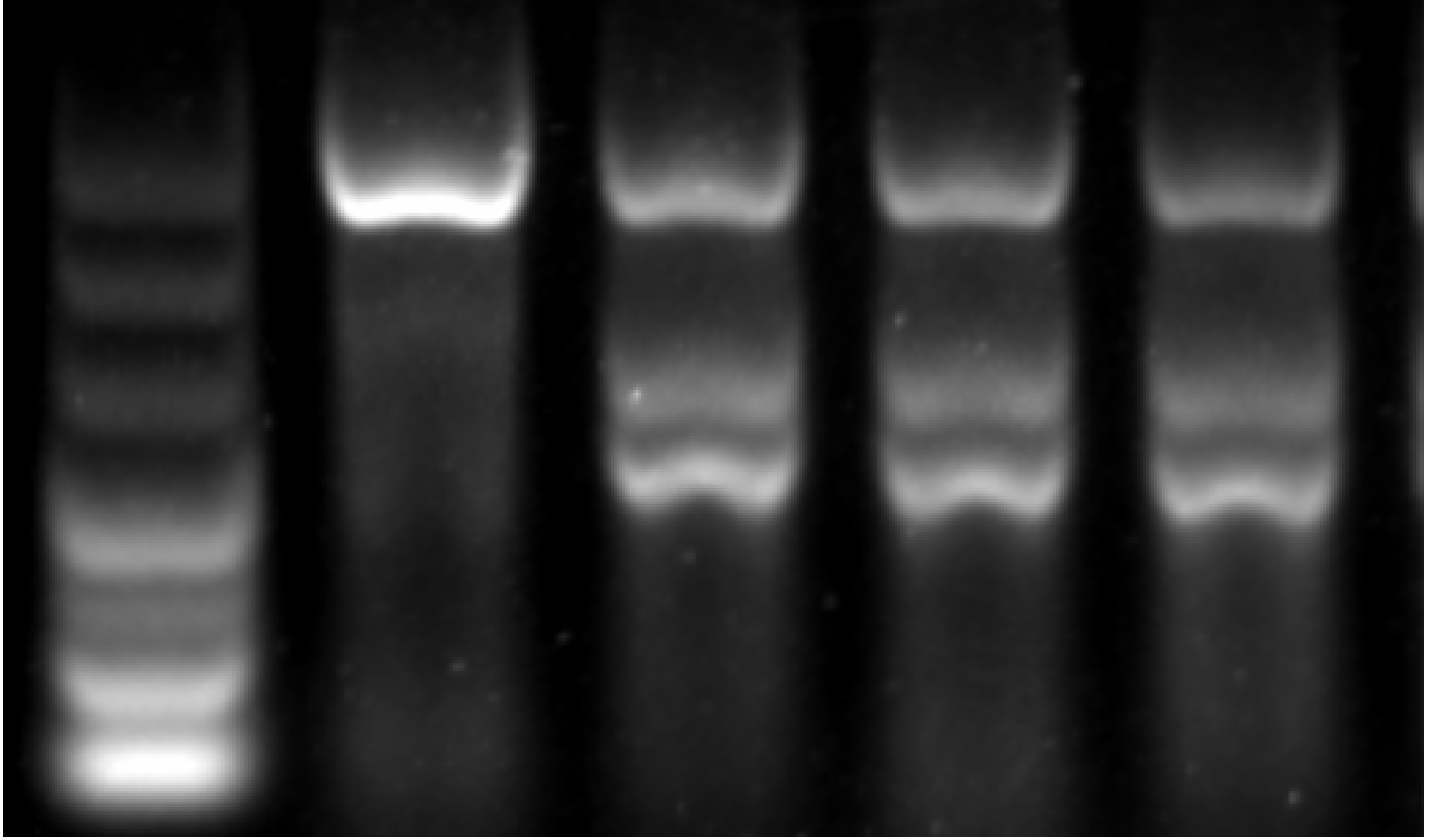

Supplement: Supplementary file 1 [file biology-12-00899-s001.zip › Original Gels/Figure 1B.png]

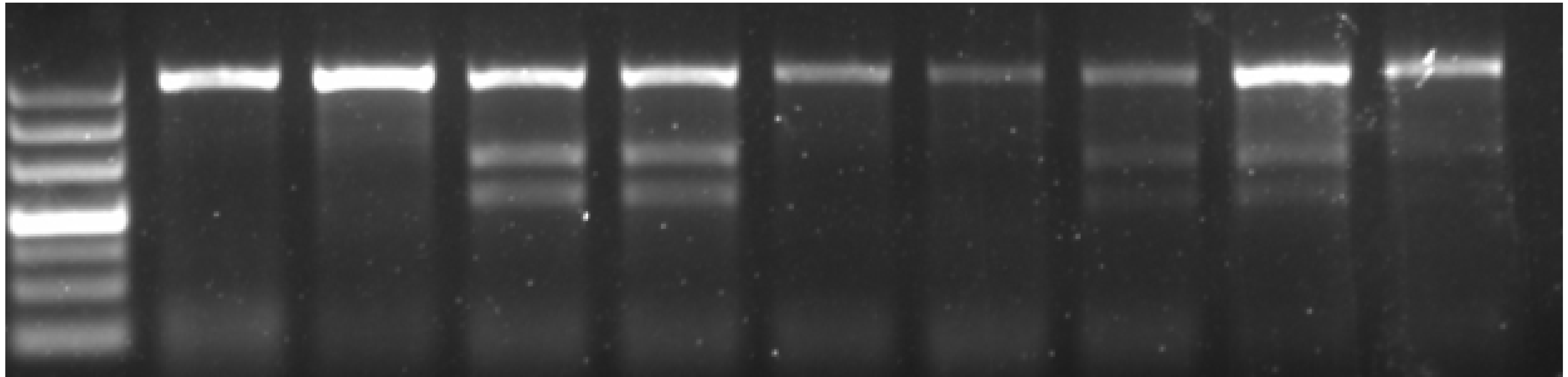

Supplement: Supplementary file 1 [file biology-12-00899-s001.zip › Original Gels/Figure 1C.png]
